# Possible relationship between esophageal dilatation and severity of *M*. *abscessus* pulmonary disease

**DOI:** 10.1371/journal.pone.0261866

**Published:** 2021-12-23

**Authors:** Hiromichi Hara, Keitaro Okuda, Jun Araya, Hirofumi Utsumi, Daisuke Takekoshi, Saburo Ito, Hiroshi Wakui, Shunsuke Minagawa, Takanori Numata, Kazuyoshi Kuwano

**Affiliations:** Division of Respiratory Diseases, Department of Internal Medicine, The Jikei University School of Medicine, Tokyo, Japan; Clinic for Infectious and Tropical Diseases, Clinical Centre of Serbia, SERBIA

## Abstract

**Objectives:**

Recently, incidence of *Mycobacterium abscessus (Mab)* pulmonary disease (Mab-PD) is increasing worldwide. We aimed to identify factors associated with severity of *Mycobacterium abscessus (Mab)* pulmonary disease (Mab-PD).

**Methods:**

All patients diagnosed as Mab-PD based on the official ATS/IDSA statement between 2017 January 1 and 2021 July 31 were included (n = 13). We reviewed medical records, bacteriological and laboratory data of the patients. Severity of lung lesions and esophageal diameters in chest CT were quantitatively evaluated. Gaffky score in the sputum was used as airway mycobacterial burden. We explored the factors associated with high CT score and high Gaffky score.

**Results:**

Maximum diameter of esophagus (MDE) in severe disease (CT score≧10) was greater than that in milder disease (CT score<10) (18.0±7.9mm, 9.3±3.1mm, respectively, p = 0.01), and MDE was well correlated with CT score (R = 0.69, p = 0.007). MDE in high mycobacterial burden group (Gaffky score ≧5) tended to be greater than that in low mycobacterial burden group (Gaffky score <5) (16.1±6.8mm, 10.1±5.5mm, respectively, p = 0.12), and MDE was well correlated with Gaffky score (R = 0.68, p = 0.009). Lung lesions were bilateral and predominant in middle or lower lobes.

**Conclusions:**

Esophageal dilatation was correlated with severity of *Mab*-PD and airway mycobacterial burden. Gastroesophageal reflux might be associated with *Mab* disease progression.

## Introduction

*Mycobacterium abscessus* (*Mab*) is one of rapidly growing non-tuberculous mycobacteria (NTM) [1]. *Mab* comprises three subspecies (*M*. *abscessus subsp*. *abscessus*, *M*. *abscessus subsp*. *massiliense and M*. *abscessus subsp*. *bolletii*.), which affects both airway and lung parenchyma [2,3]. In comparison to *M*.*avium complex(MAC)*, *Mab* is less commonly isolated from respiratory specimens [4], hence clinical characteristics of *Mycobacterium abscessus (Mab)* pulmonary disease (Mab-PD) remains to be elucidated.

Recently, incidence of Mab-PD is increasing worldwide including Japan [4,5]. Indeed, the incidence rate for Mab-PD increased from 0.1 cases in 2001 to 0.5 cases per 100000 person-years in 2014 in Japan [5]. Mab-PD often results in poor clinical outcome due to broad drug resistance of the mycobacteria [6,7]. Thus, Mab-PD is becoming an important public health problem, and there is an urgent need for elucidating clinical characteristics and the risk factors for Mab-PD exacerbation to diagnose in early stage and prevent progression of the disease.

A variety of comorbidities (bronchiectasis, obstructive lung diseases including asthma and COPD, malignancies, rheumatoid arthritis, gastroesophageal reflex and so on) and conditions (steroid use, inhaled corticosteroid use, low body weight) were considered to be associated with pulmonary infection of NTM [4,8–13]. Those underlying clinical conditions might be associated with not only increased incidence of NTM-PD but also progression of the disease. However, reported papers do not precisely evaluate the association between underlying clinical conditions and disease severity of Mab-PD. In this study, we investigated characteristics of Mab-PD patients and attempted to identify the factors associated with disease severity and airway mycobacterial burden.

## Methods

### Study subjects

This study is a retrospective cohort study of patients diagnosed as Mab-PD based on the official ATS/IDSA statement at Jikei University Hospital from January 1, 2017 (01/01/2017) to July 31, 2021 (07/31/2021). Patients were eligible for the study if they met the following inclusion criteria 1) Age ≥ 20 years old, 2) patients diagnosed as Mab-PD based on the official ATS/IDSA statement [1].

A total of 13 patients were included in this study. This study was approved by the Ethics Committee of Jikei University (33–219 (10836)), and adhered to the tenet of medical ethics. Based on the ethical guidelines of Jikei University, informed consent was not necessary for this retrospective study, and we performed opt-out consent on the website of our hospital. All data were fully anonymized after we collected data from medical records. All data was anonymized prior to statistical analysis. There are ethical restrictions on sharing de-identified data set since the data contain potentially identifying or sensitive patient information. Data from this study are available upon request (Hiromichi Hara, E-mail: hirohara@jikei.ac.jp or ethics committee of Jikei University, E-mail: crb@jikei.ac.jp). Patients’ medical records during 2017 (01/01/2017) to July 31, 2021 (07/31/2021) were accessed. Chest computed tomography (CT) was performed when obtained respiratory samples proved to be culture-positive for *Mab*. The chest CT findings of these patients were evaluated by two specialists of the Japanese Respiratory Society (H.H. and O.K.).

### Diagnosis of Mab-PD

Respiratory samples from patients with clinical suspicion of NTM pulmonary disease were cultured on Ogawa media, and species of cultured mycobacteria were determined by mass spectrometry or DDH commercially (SRL, Inc. laboratory, Tokyo, Japan). Diagnosis of Mab-PD was made based on the official ATS/IDSA statement: diagnosis, treatment, and prevention of nontuberculous mycobacterial diseases [1].

### Data collection

We retrospectively reviewed the medical records of the enrolled patients. Clinical characteristics including age, sex, BMI, smoking history, commobidities, underlying lung disease, symptoms of increased sputum, and Gaffky score in sputum were investigated. Maximum number of Gaffky score was recorded. CT score was calculated based on a scoring system for MAB-PD in a previous report [14]. A total score of 30 was allocated for five types of lung lesions including bronchiectasis (9 points), bronchiolitis (6 points), cavity (9 points), nodule (3 points), and consolidation (3 points) depending on severity of lung lesions. Maximum diameter of esophagus (MDE) was measured on axial Chest CT images (5mm slice), and the longest distance of the esophageal air was measured without touching the wall as reported in a prior study [15]. We defined radiologically severe disease as CT score≧10 and milder disease as CT score<10. We also defined bacteriologically severe group as Gaffky score≧5 and milder disease as Gaffky score <5. MDE≧15mm was considered Esophageal dilatation [16] according to the previous report.

### Statistical analysis

Student’s t-tests, or Fisher’s exact tests were performed to compare clinical indices of radiologically severe disease (CT score≧10) and milder disease (CT score<10). Clinical indices of bacteriologically severe group (Gaffky score≧5) and milder disease (Gaffky score <5)were also compared. Regression analysis were performed to estimate the relationship between MDE and CT score, MDE and Gaffky scale. A two-sided p<0.05 was considered statistically significant. All statistical analyses were performed with StatView, version 5 (SAS Institute Inc., Cary, NC, USA).

## Results

### Clinical characteristics of Mab-PD (Table 1)

Characteristics of Mab-PD patients (n = 13) were shown in Table 1. Four patients (30.8%) had obstructive lung disease, and 3 patients (23.1%) used ICS. Six patients out of 12 patients (50%) were positive anti-GPL-core IgA antibody, and sputum culture of 3 patietns (23.1%) were positive for other NTM or for aspergillus.

**Table 1:** Clinical characteristics of all patients (n = 13).

	Total (n = 13)
Age (years)	61.4±16.5
Male (%)	4 (30.8)
BMI	19.5±3.2
History of smoking (%)	4 (30.8)
malignancy(%)	3 (23.1)
GERD(%)	4 (30.8)
Autoimmune diseases(%)	2 (15.4)
Use of steroid or immunosuppressant(%)	4 (30.8)
Obstructive lung diseases(%)	4 (30.8)
Use of inhaled corticosteroid(%)	3 (23.1)
increased sputum	8 (61.5)
Maximum number of Gaffky scale	3.6±2.8
Gaffky scale ≧5(%)	4 (30.8)
CT score	7.4±3.4
Maximum diameter of esophagus(mm)	12.0±6.3
Alb(g/dl)	3.9±0.4
CRP(mg/dl)	1.4±2.6
positive anti-GPL-core IgA antibody	6(50)
	unknown n = 1
positive culture of other NTM in sputum	3 (23.1)
positive culture of other aspergillus in sputum	3 (23.1)

BMI;Body Mass Index.

GERD;Gastroesophageal Reflux Disease.

NTM;Non-tuberculous mycobacteria.

### Comparison of clinical characteristics of patients with severe disease and milder disease (Table 2)

**Fig 1:**
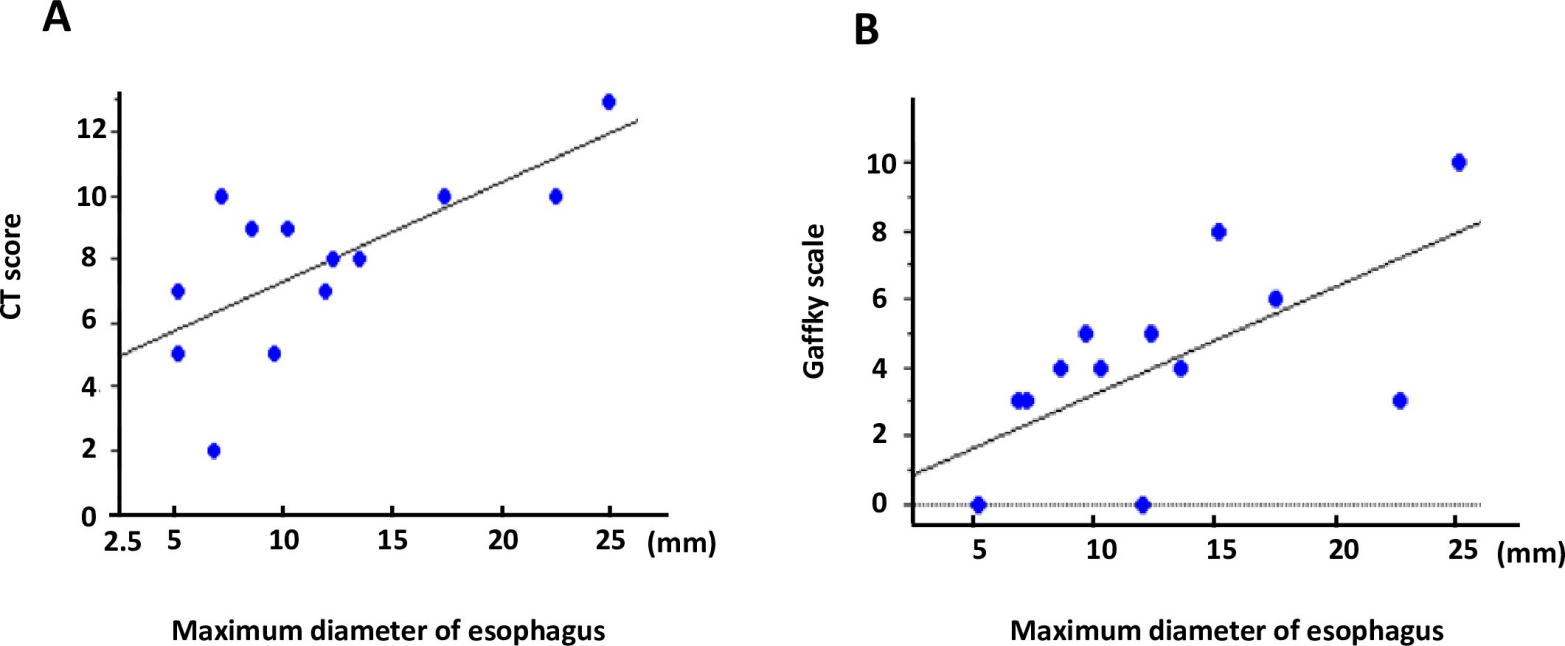
Correlation of CT score(A), gaffkey score (B) and maximum diameter of esophagus (MDE) Correlation of CT score and maximum diameter of esophagus (MDE) is shown in A. Correlation of gaffky score and MDE is shown in B.

According to previous paper, severity of lung disease was scored by means of CT evaluation [14]. Mab-PD patients were divided into two groups according to the severity of the lung disease (CTscore≧10, and CT score<10). Maximum diameter of esophagus (MDE) in severe disease (CT score≧10) was greater than that in milder disease (CT score<10) (18.0±7.9mm, 9.3±3.1mm, respectively, p = 0.01). Maximum number of Gaffky score in severe disease (CT score≧10) tended to be greater than that in milder disease (5.5±3.3, 2.8±2.2, respectively, p = 0.1). MDE was well correlated with CT score (R = 0.69, p = 0.007, Fig 1A).

**Table 2:** Comparison of clinical characteristics of patients with severe disease and milder disease.

	CTscore≧10 (n = 4)	CTscore<10 (n = 9)	p value
Age (years)	68.5±8.2	58.2±18.6	0.32
Male (%)	2 (50)	2 (22.2)	0.53
BMI	17.4±3.7	20.5±2.6	0.11
History of smoking (%)	1 (25)	3 (33.3)	>0.99
malignancy(%)	1 (25)	2 (22.2)	>0.99
GERD(%)	2 (50)	2 (22.2)	0.53
Autoimmune diseases(%)	0 (0)	2 (22.2)	>0.99
Use of steroid or immunosuppressant(%)	1 (25)	3 (33.3)	>0.99
Obstructive lung diseases(%)	2 (50)	2 (22.2)	0.53
Use of inhaled corticosteroid(%)	1 (25)	2 (22.2)	>0.99
increased sputum	1 (25)	7 (77.8)	0.22
Maximum number of Gaffky scale	5.5±3.3	2.8±2.2	0.1
Gaffky scale ≧5(%)	2 (50)	2 (22.2)	0.53
CT score	10.8±1.5	6.67±2.3	0.008
Maximum diameter of esophagus(mm)	18.0±7.9	9.29±3.1	0.01
Alb(g/dl)	4.0±0.5	3.9±0.4	0.83
CRP(mg/dl)	1.4±2.6	1.4±2.8	0.99
positive anti-GPL-core IgA antibody	2 (50)	4 (50)	>0.99
		unknown n = 1	
positive culture of other NTM in sputum	1 (25)	2 (22.2)	>0.99
positive culture of other aspergillus in sputum	1 (25)	2 (22.2)	>0.99

BMI;Body Mass Index.

GERD;Gastroesophageal Reflux Disease.

NTM;Non-tuberculous mycobacteria.

### Distribution and CT pattern of parenchymal abnormality in Mab-PD (Table 3)

Distribution and CT pattern of parenchymal abnormality in Mab-PD were shown in Table 3. Bilateral bronchiectasis and bronchiolitis in middle or lower lobes were predominant. Cavity and nodules were rare in this study.

**Table 3:** Distribution and CT pattern of parenchymal abnormalities in Mab-PD (n = 13).

					Right lung			Left lung		
CT pattern	CT score(average)	Unilateral	Bilateral	Upper	Middle	Lower	Upper	Lingular	Lower	total (Max 78)
Bronchiectasis	2.5	1	9	4	10	6	1	7	7	35
Bronchiolitis	2.9	1	10	4	8	9	1	8	8	38
Cavity	0.8	1	1	0	0	1	0	0	2	3
Nodules	0.3	4	0	1	0	2	0	0	1	4
Consolidation	0.9	3	6	2	4	7	2	3	4	22

### Clinical characteristics of patients with high mycobacterial burden in sputum (Table 4)

**Fig 2:**
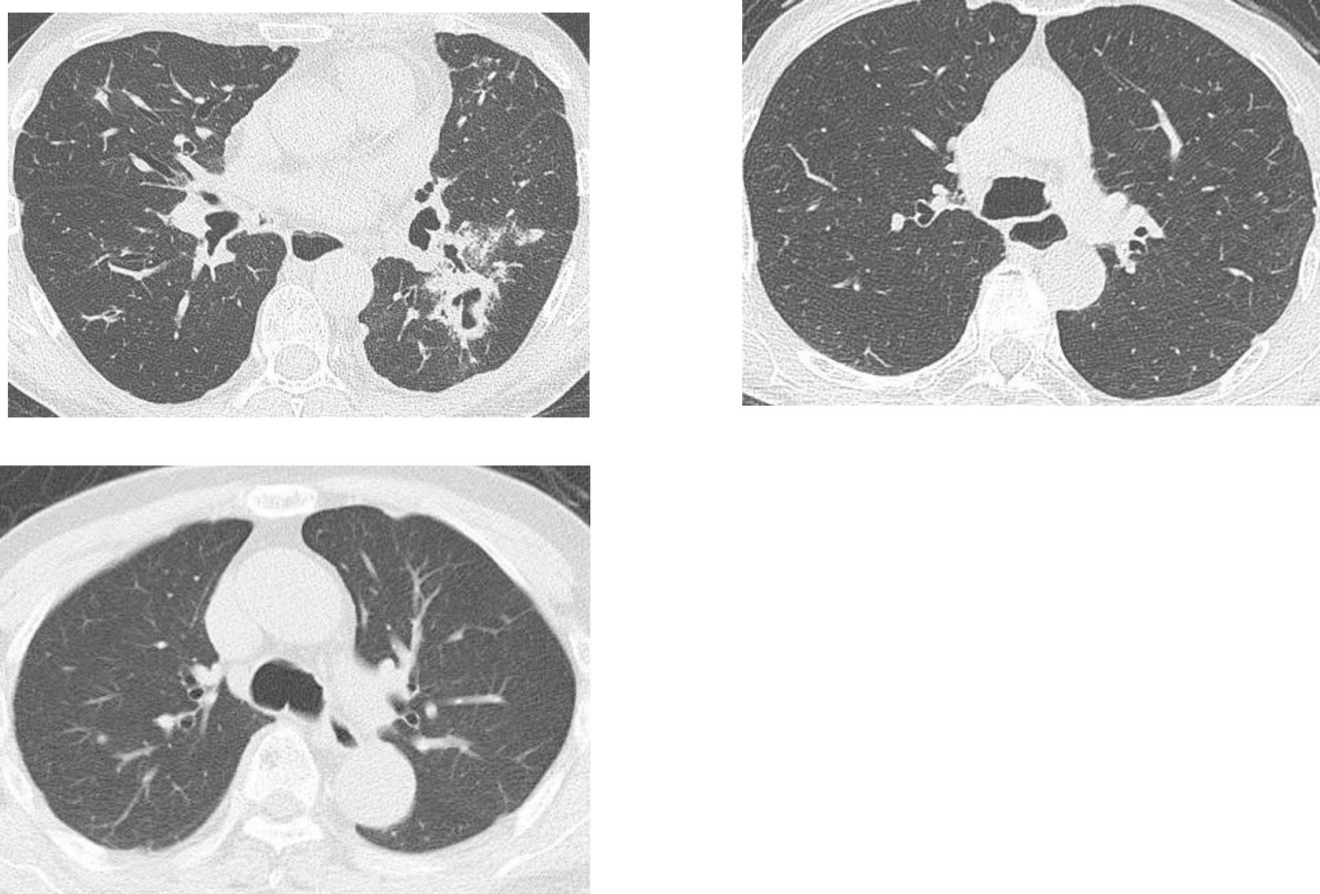
Typical cases of esophageal dilatation. CT images of three cases whose maximum diameter of esophagus is larger than 15mm were shown.

**Fig 3:**
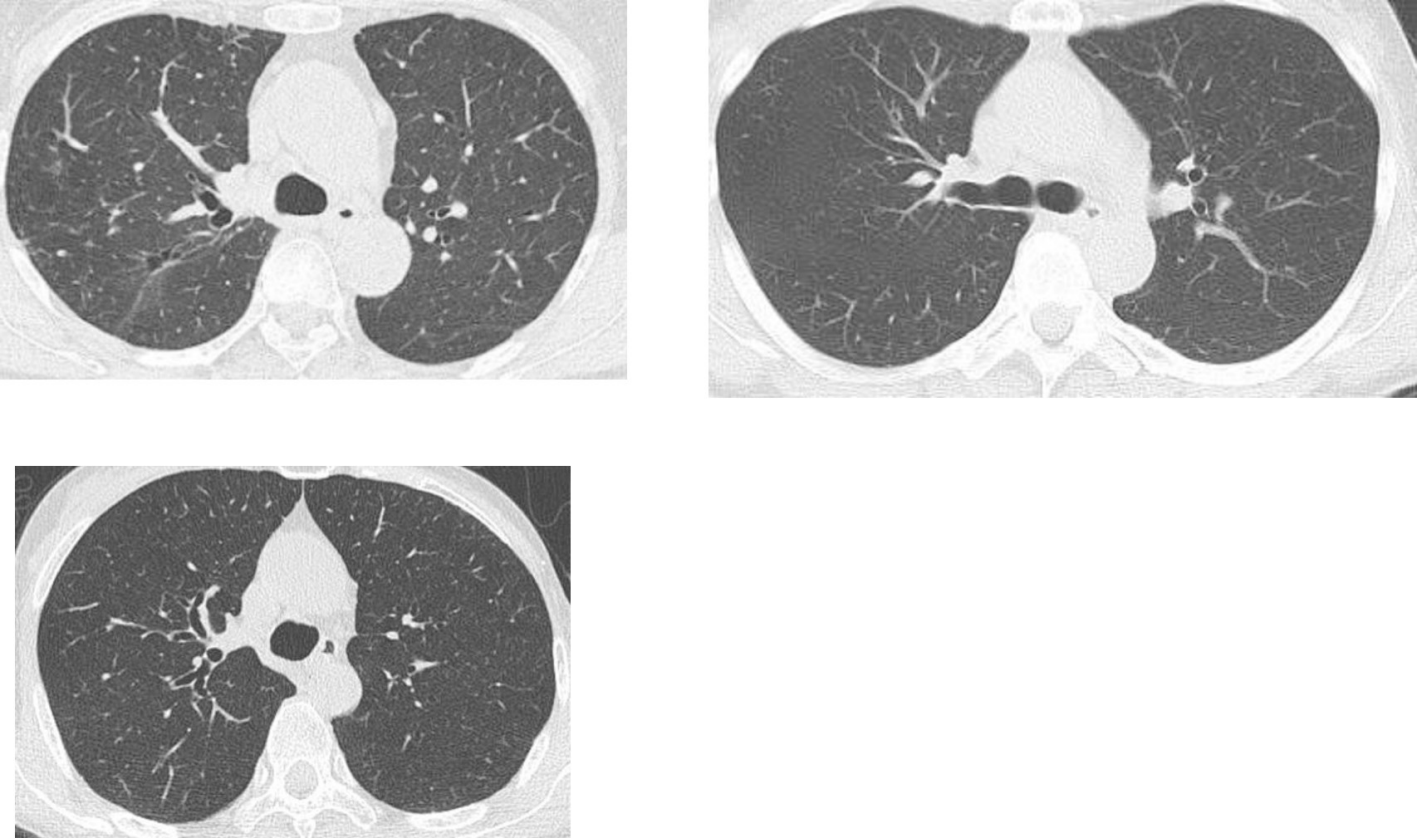
Typical cases of normal esophageal diameter. CT images of three cases whose maximum diameter of esophagus is less than 10 mm were shown.

Clinical characteristics of patients with high mycobacterial burden in sputum was shown in Table 4. Obstructive lung disease and use of inhaled steroid, well-known risk factors for NTM were associated with high mycobacterial burden. MDE in high mycobacterial burden group (Gaffky score ≧5) tended to be greater than that in the low mycobacterial burden group (Gaffky score <5) (15.0±5.8mm, 9.8±5.3mm, respectively, p = 0.03), and MDE was well correlated with Gaffky score (R = 0.68, p = 0.009 Fig 1B). We showed CT scans of typical cases of esophageal dilatation in Fig 2, and those of normal esophageal diameter in Fig 3.

**Table 4:** Clinical characteristics of patients with high bacterial burden in sputum.

	Gaffky≧5 (n = 4)	Gaffky<5 (n = 9)	p value
Age (years)	56.8±16.7	63.4±16.9	0.52
Male (%)	3 (75)	1 (11.1)	0.05
BMI	20.9±4.5	18.9±2.5	0.32
History of smoking (%)	0 (0)	4 (44.4)	0.23
malignancy(%)	1 (25)	2 (22.2)	>0.99
GERD(%)	2 (50)	2 (22.2)	0.53
Autoimmune diseases(%)	0 (0)	2 (22.2)	>0.99
Use of steroid or immunosuppressant(%)	1 (25)	3(33.3)	>0.99
Obstructive lung diseases(%)	4 (100)	0 (0)	0.001
Use of inhaled corticosteroid(%)	3 (75)	0 (0)	0.01
increased sputum	3 (75)	5(55.6)	>0.99
Maximum number of Gaffky scale	6.5±2.4	2.3±1.8	0.005
CT score	9.0±3.4	7.4±2.6	0.38
Maximum diameter of esophagus(mm)	16.1±6.8	10.1±5.5	0.12
Alb(g/dl)	3.8±0.48	4.0±0.44	0.49
CRP(mg/dl)	1.9±2.5	1.2±2.8	0.68
positive anti-GPL-core IgA antibody	2(50)	4 (50)	>0.99
		unknown n = 1	
positive culture of other NTM in sputum	1 (25)	2 (22,2)	>0.99
positive culture of other aspergillus in sputum	2(50)	1 (11.1)	0.2

BMI;Body Mass Index.

GERD;Gastroesophageal Reflux Disease.

NTM;Non-tuberculous mycobacteria.

## Discussion

In this study, we demonstrated that (1)Maximum diameter of esophagus (MDE) in radiologically severe disease (CT score≧10) was greater than that in milder disease (CT score<10) and MDE was well correlated with CT score. (2) MDE in high mycobacterial burden group (Gaffky score ≧5) tended to be greater than that in low mycobacterial burden group (Gaffky score <5), and MDE was well correlated with Gaffky score. Accordingly, esophageal dilatation can be associated with severity of Mab-PD and airway mycobacterial burden. It is also likely that gastroesophageal reflux might be associated with *Mab* disease progression.

Gastroesophageal reflux disease (GERD) is one of the well-known risk factors for NTM-PD [17]. Since NTM can survive in acid conditions, GERD followed by aspiration of refluxed gastric contents containing NTM is one of considerable mechanisms of NTM-PD [18]. Since significant relationship between the presence of GERD and esophageal dilatation (ED) in the CT scan was shown in a previous study [19], we measured diameters of esophagus to detect subclinical or undiagnosed GERD. Both radiological and bacteriological disease severities were significantly correlated with MDE in this study, suggesting that subclinical GERD is highly associated with pathogenesis of *Mab* progression of pulmonary disease. In consistent with our results, Koh et.al previously reported that NTM patients with GERD were more likely to have a positive sputum smear for acid-fast bacilli and were affected more lobes than patients without GERD [17]. Involvement of Mab-PD lesions were bilateral and predominant in middle or lower lobes in the present study, which may reflect the participation of GERD-associated aspiration in progression of lung lesions. Since CT is routinely performed to evaluate lung lesions of Mab-PD in almost all patients in Japan, MDE might be a useful tool to detect subclinical GERD without additional invasive procedure. In patients with increased MDE, improving intestinal motility, and Semi-Fowler’s positioning during night might have some effect to attenuate disease progression of Mab-PD by preventing aspiration of swallowed mycobacteria or contaminated water or food.

This study has several limitations. Firstly, due to low prevalence of *Mab* infection, the sample size was small. We could not perform multivariate analysis. Confounding factors might influence the results of this study. However, esophageal dilatation evaluated by MDE showed strongest association with disease severity among known risk factors for NTM in monovariate analysis. Although larger studies are necessary to confirm our results, MDE could be a useful and simple biomarker for Mab-PD exacerbation. Secondly, patients’ population of this study might be different from patients in previous reports. While patients classified as upper lobe cavitary form were included in other studies, there were no such patients in this study. Upper lobe cavitary form may not to be associated with GERD, suggesting that risk factors for Mab-PD can be different in those patients. Finally, we did not determine subspecies of *Mab* and clinical characteristics can be different among the subspecies. Although previous studies demonstrated that CT findings of *M massiliense* disease overlap with those of *M abscessus* disease, further studies are needed to precisely evaluate the difference among *Mab* subspecies.

## Conclusions

Esophageal dilatation was associated with severity and airway mycobacterial burden of *Mab* infection. It is likely that unaware gastroesophageal reflux can be causally linked to *Mab* disease progression.

## Abbreviations

*Mab*: *Mycobacterium abscessus*
*MAC*: *M*.*avium complex*
NTM: non-tuberculous mycobacteria
PD: Pulmonary disease
GERD: Gastroesophageal reflux disease
